# COVID-19 preparedness and social dynamics in a Sub-Saharan Africa country, Benin

**DOI:** 10.1093/heapro/daac105

**Published:** 2022-08-24

**Authors:** Alessia Maccaro, Davide Piaggio, Marius Vignigbé, Alexander Stingl, Leandro Pecchia

**Affiliations:** School of Engineering, University of Warwick, Library Road, CV47AL Coventry, UK; Institute of Advanced Studies, University of Warwick, Library Road, CV47AL Coventry, UK; School of Engineering, University of Warwick, Library Road, CV47AL Coventry, UK; Département de Sociologie-Anthropologie, University of Abomey-Calavi, Abomey-Calavi, Benin; Institute of Advanced Studies, University of Warwick, Library Road, CV47AL Coventry, UK; School of Engineering, University of Warwick, Library Road, CV47AL Coventry, UK; School of Engineering, Università Campus Bio-Medico di Roma, Via Álvaro del Portillo, Roma, Italy

**Keywords:** COVID-19, preparedness, Sub-Saharan Africa, bioethics, health policy

## Abstract

This project aims to assess and analyse the perception and impact of the COVID-19 pandemic in Benin. The applied research methodology was interdisciplinary and combined field studies that used ethnographic and social research methods with coding and data analysis, leading to theoretical dilemmas, which were analysed from the viewpoint of bioethical reflection. Furthermore, biomedical engineering approaches were used to assess the preparedness to COVID-19. Despite the preparedness to COVID-19 due to the promoted governmental measures, a peculiar management of the pandemic emerged. The latter, although noteworthy, did not overcome the typical challenges of medical locations in low-resource settings. This, together with the controversial spread of information and local beliefs, caused significant economic and social consequences, exceeding the benefits related to the containment of the virus. This research highlights how the emotion of fear, in this specific situation, was herald of dramatic consequences, rather than having a heuristic and empowering effect.

## INTRODUCTION

The SARS-CoV-2 disease (COVID-19) was declared a pandemic by the World Health Organization (WHO) on 11 March 2020, after a first wave of infections was reported in China at the end of 2019. Since then, it has become a global emergency and has continued spreading across the world. In Benin the first case was declared on 16 March 2020 (Osseni, 2020).

As of 25 November 2020, Africa counted a total of 2.1 million confirmed cases and almost 51 thousand deaths, considerably less than other continents. Europe, in fact, totalled over 16 million cases and 370 thousand deaths, and North America over 15 million cases and 398 thousand deaths (Musa *et al.*, 2020).There have been different attempts to explain this uneven distribution and morbidity of COVID-19, globally, including: the alleged genetic immunity of some ethnicities (Musa *et al.*, 2020); the theory sustaining that some climatic conditions are inhospitable for SARS-CoV-2 (Nguimkeu and Tadadjeu, 2020); the relatively young age of the population (Musa *et al.*, 2020; Nguimkeu and Tadadjeu, 2020; WHO, 2020); the lower population density and urbanization rates of Sub-Saharan Africa (Nguimkeu and Tadadjeu, 2020). Other theories claimed that the previous epidemics (e.g. Ebola) had strengthened low- and middle-income countries (Keck, 2015), making them more prepared for such outbreaks (Ayebare *et al.*, 2020; Kapata *et al.*, 2020; Leach, 2020; Musa *et al.*, 2020).Finally, there are the conspiracy theorists and deniers arguing that the pandemic is only affecting some world regions due to specific political reasons (Mwaura, 2020).

Indeed, it is necessary to remember that the screening activities are not always comparable among countries (Musa *et al.*, 2020). Thus, it is likely that the actual number of cases and deaths detected in the Global South is far from the numbers reported (Nakkazi, 2020).

As of 24 May 2021, Benin has reported 31 active cases for a total of 8025 confirmed cases including 7893 cured and 101 deaths (https://www.gouv.bj/coronavirus/).

The pandemic has affected healthcare systems around the world, resulting in low-resource settings (LRSs) becoming the general norm, i.e. environments lacking means, specific knowledge, specialized personnel, medical devices and drugs and with inappropriate medical locations. This has important ethical implications (e.g. the definition of scarce resource allocation criteria). In fact, while this situation was already familiar to low- and middle-income countries, COVID-19 has overwhelmingly created LRS conditions in high-income regions, such as Europe (Pecchia *et al.*, 2020).

Nonetheless, significant differences among countries remain and even become more acute with regard to the social consequences of the pandemic. To this regard, in May 2021, Johri *et al.* (Johri *et al.*, 2021) has recently (May 2021) discussed the social consequences of COVID-19 on poor and vulnerable groups in India, arguing that in times of pandemic, the infection, prevention and control measures (e.g. confinement) should be equipoised in respect to their impact on local societies, minorities and economies.

For this reason, given the extraordinary social impact of the current pandemic, it was considered urgent to investigate the causes, starting from a survey on the social perception of COVID-19.

This article presents the results of this project, conducted by a multidisciplinary team involving sociologists, ethicists and biomedical engineers from the University of Warwick (UK) and of Abomey-Calavi (Benin), in order to evaluate the population’s perception in respect to COVID-19, as well as the governmental measures and preparedness, also concerning the Beninese healthcare system.

## METHODOLOGY

**Fig. 1:**
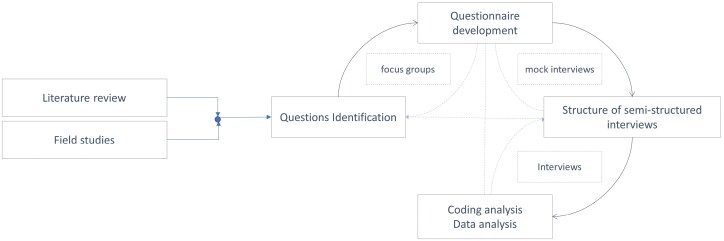
The various phases, applied methods, main objectives and outcomes of the project.

The inductive method, leveraging several field observations to generate theories (Goddard and Melville, 2004), was applied throughout this project. Figure 1 shows the phases of the project along with their main objectives, applied methods and outcomes. In particular, purposive sampling (Pope and Mays, 1995; Ritchie *et al.*, 2013), i.e. a non-random technique consisting in the deliberate choice of participants by the researcher, according to the information they can provide based on their knowledge or experience (Etikan *et al.*, 2016), was selected and applied for any method requiring sampling.

This project largely relies on field observations, situational and field notes analysis, as well as semi-structured interviews to capture the opinions and perception on preparedness of part of the Beninese population in contact with the public and local communities. The cross-analysis *a posteriori* of such collected data allowed the generation of theories that could be compared with the relevant literature (Figure 1).

### Literature review

Relevant scientific literature was retrieved from databases such as Scopus using related keywords. Further literature was found applying a snowballing technique to the retrieved articles (Marshall, 1998). Gray literature (e.g. the governmental websites, COVID-19 data, etc.) was retrieved by searching on Google.

### Field notes

Field notes taken during four field studies were used to support the analysis of the engineering aspects and of the preparedness of the healthcare locations, along with the related publications (Pecchia, 2017; Piaggio *et al.*, 2019; Di Pietro *et al.*, 2020). Three of the aforementioned field studies took place in Benin (April 2017, January 2018 and November 2019) and one in Uganda (October 2019). During these field studies, the assessment of the conditions of medical devices and medical locations took place, as well as the ethnographic reconstruction of the local Beninese culture. Furthermore, issues related to the negotiation between local cultural particularism and the universalism of regulations and human rights, which are included in intercultural bioethics, have already been investigated and reported (Maccaro, 2019a, b, Maccaro *et al.*, 2020).

### Semi-structured interviews

The first draft of the two semi-structured interviews, i.e. sociological and engineering, were created after a focus group with experts of ethics, bioethics, biomedical and clinical engineering, regulations and standards and pandemic preparedness. The validity of the two surveys was investigated in two stages through two pilot tests. Firstly, they were reviewed among the participants to the focus group for disambiguation and correction of content and language. Later on, it was tested with online semi-structured interviews, recruiting Beninese scholars not included in the former focus group. This was used as a further refinement of the semi-structured interview. The final questionnaire was written in two languages, i.e. English and French, as Benin is francophone and was held in French.

Interviewees were identified among workers at the Beninese Ministry of Health, biomedical engineers and technicians, healthcare workers, the police, anthropologists, public health experts and civilians.

The full texts of the semi-structured interviews (available upon reasonable request by contacting the authors) were coded using NVivo 12-pro by two of the authors and were reviewed by a third author. The recurrency of the identified nodes (i.e. themes) along with their interrelations was analysed to investigate and retrieve meaningful data.

The study received full ethics approval by the University of Warwick Ethical BSREC Committee (BSREC 140/19-20). Informed consent was sought before the start of the interviews.

### On ‘intersecting cultural domains’

Precisely, the goal of our study was not to fall back into anything resembling those debunked patterns of casting local, indigenous health cultural domains as ‘primitive’ and ‘obstacles to Western modern practices’. We consider that what was once called ‘*emic* perspectives’ matters profoundly (*emic* meaning here the internal elements and their internal consistency, resilience and rationality as functional and functioning framework; *etic*, on the other hand, refers to an outsider’s framework in the situation of cultural interpretation and interaction). Quiroz and van Andel (Quiroz and van Andel, 2018) underlined the social role of the utilization of plants in Benin, which cannot be simplified to the imperial dychotomies of ‘Euromodern vs. primitive’ typical of the 20th century. Methodologically, this requires the research to ‘learn to see otherwise’ (Decoteau, 2017a).

Similarly, Carolyn Sargent, in her ground-breaking medical anthropological studies of Benin, begs the question how ‘collaborative relationships’ are possible in practical terms (Chary and Sargent, 2016). Sargent allows us to see an even more interesting tension (Stroeken *et al.*, 2017) than the merely epistemological and systemic conflict between Western (allopathic) medicine and any non-Western medicine.

These and related tensions are in the case of newly emerging diseases often expressed by indigenous populations in the Global as well as non-Western migrant populations in terms of a cultural ascription, as Decoteau (Decoteau, 2017b) shows in the case of autism, as ‘Western’ or ‘Global Northern disease’.

Accepting that it is not a single cultural factor that can lead to conflict and, thus, to problematic outcomes, such as we describe in the case of COVID-19, we take inspiration from Behague *et al.* (Behague *et al.*, 2008), that a proper understanding of the complexities at work and accepting cultural differences can also lead to *transformative agency.*

In other words, instead of merely looking at a problem presented by such complexity and finding a technical solution to solve or overcoming the problem by negating a cultural factor, find *generative opportunities* within the complexities of the problem itself [as also described as a methodological paradigm in (Stingl 2015)] (Damien *et al.*, 2020).

Along those lines, we find further inspiration in social research approaches that are inclusive of ‘seeing otherwise’: drawing also on the Bourdieu (Behague *et al.*, 2008), as well as the biopolitical critique of Michel Foucault and the methodological purview of Critical Realism, in her seminal research on HIV, masculinity, indigeneity and normalizing, neoliberal biomedical rationalities in South Africa, Claire Decoteau has illustrated how tensions between *emic* and *etic* perspectives lead to conflicts.

## RESULTS

The results presented in the following sections are a critical analysis of the semi-structured interviews, the retrieved literature and our field notes. Unfortunately, due to COVID-19 restrictions, it was not possible to hold all the scheduled interviews. Consequently, a total of 13 interviews were held. Among the interviewees, 4 (30.8%) were healthcare workers, 3 (23.1%) public health experts, 1 (7.7%) hotel manager, 1 (7.7%) teacher, 1 (7.7%) hygienist, 1 (7.7%) anthropologist and 1 (7.7%) policeman.

Nonetheless, the results are corroborated by a thorough analysis also referring to the relevant literature and field notes.

### Literature review: the main governmental provisions

Online Resource 1 reports the main governmental provisions in Benin.

### Engineering semi-structured interviews and field notes

Benin, as the rest of Africa, and most of LRSs, has been and is facing different challenges, including:

(1) The lack of COVID-19 test kits, with the consequent under-detection of cases (Paintsil, 2020).
(2) The non-ideal screening of travellers and the different quality of screening dependent on the point of entry (Paintsil, 2020).
(3) The shortage of personal protective equipment and of healthcare workers and infrastructure (Dubbink *et al.*, 2020; Finn and Kobayashi, 2020; Osseni, 2020; Post *et al.*, 2020).
(4) The fear of stigmatization preventing ill people from going to the hospital (Dubbink *et al.*, 2020; Post *et al.*, 2020).
(5) Lack of access to adequate healthcare, housing and sanitation (Finn and Kobayashi, 2020; Post *et al.*, 2020).
(6) Overburdened healthcare settings (Dubbink *et al.*, 2020).
(7) Lack of adequate centres equipped for the treatment of COVID-19 (Dubbink *et al.*, 2020; Osseni, 2020; Paintsil, 2020; Post *et al.*, 2020).

In particular, during our field studies in Benin we could assess the conditions of several hospitals, both private and public (Piaggio *et al.*, 2019; Di Pietro *et al.*, 2020). Indeed, a lack of essential medical devices and the relative spare parts, of a good health technology management system, of preventive maintenance and of the tools needed for it (e.g. electrical safety analysers and other analysers), of specialized doctors and biomedical engineers/technicians, as well as the harsh environmental conditions and unstable power supply, contributed to creating a very challenging environment even before the COVID-19 pandemic started (Di Pietro *et al.*, 2020). The situation was exacerbated with the start of the pandemic, as a more specific set of medical devices (e.g. ventilators), analysers (e.g. gas flow analysers) and expertise were needed. For detailed information on the presence of essential medical devices and their status (i.e. functioning or broken), please refer to (Di Pietro *et al.*, 2020).

From the interviews, it emerged that although there is provision for some medical devices and equipment, the new logistics commission that oversees this does not communicate with the existing actors nor provides instructions or training.

The interviewee insisted on the cruciality of the training of paramedics, healthcare workers and biomedical engineers/technicians about the new medical devices and technologies in order to

‘[…] ensure proper use and sustainability of the investment’ Health Inspector, Female, 51 years old

Finally, the interviewee stated that further technical actions are required, suggesting that putting up tents for the triaging and management of COVID-19 patients could be of great help.

### Sociological semi-structured interviews

Twelve people, 2 (16.67%) females and 10 (83.33%) males, were interviewed. Their average age was 44, and their professional/expertise sectors are reported above.

The coding analysis resulted in the individuation of 7 macrothemes, further divided into 45 themes. Online Resource 2 presents the themes and their hierarchies. The discussed topics were: Perception of the disease (42%), Disease management (25%), Governmental measures (20%), Traditional medicine (7%), Screening (4%), Pharmacological treatment (2%) and Isolation (1%).

#### Governmental measures

Since the WHO declaration of a state of pandemic, the Beninese government has distinguished itself for the implementation of an appreciable preventive strategy. In fact, it promptly started disseminating the first guidelines for the population, mobilizing international funds and human resources, requisitioning hotels to allow travellers to self-isolate and strengthening the health and police system.

Indeed, Benin, like many African countries, welcomed the technical and financial support of the WHO, which allowed it to prepare adequately for the pandemic.

‘This translates into technical support (from the point of view of standards of care, installation of prevention devices, etc.) and financial. The WHO advises countries and mobilizes the resources of financial partners (World Bank, Canadian, Japanese funds, etc.), coordinates their interventions (weekly meetings of all partners), advocates, etc. without a mechanism of coercive power over the countries. The role of the WHO is much more in support than in decision-­making […]’—Epidemiologist, Male, 58 years old.

From a management point of view, the decision of establishing a sanitary cordon among the affected areas was promptly taken with the aim of reducing travels among the countries and regions most at risk. Furthermore, in order to avoid gatherings, it was soon decided to close the places of worship and to reduce the number of passengers for buses and taxis, and to impose the use of masks, even outdoor.

From a clinical point of view, the government soon aligned itself with the WHO indications regarding the preventive strategy and hygiene measures, which resulted in a non-negligible overall improvement in the equipment and hygiene conditions of hospitals and workplaces. Furthermore, the Beninese government soon decided to follow international guidelines regarding the tests for the screening of symptoms and the treatment. The former (i.e. PCR-TDR) are only performed in big cities, which can be very far from rural areas, apart from small teams that carry out home screening, or by telephone through a call centre set up for this purpose. As for the treatment, it is mainly based on chloroquine, azithromycin and vitamin C to strengthen the immune system. The communication of positivity to COVID-19 takes place after 72–96 hours on the phone or in person by an interdisciplinary team, which sometimes also includes a psychologist.

The complexity of this disease, being surrounded by a veil of uncertainties, highlighted the essential role of psychologists. In fact, during this pandemic, in Benin, psychologists were recognized as the vital workers, who were in charge of managing the communication of the positive outcome of the test, guaranteeing privacy and managing fear.

#### Disease management

The drug treatment is pre-emptively administered to those who have symptoms and are waiting for test results. In case of confirmation of positivity, it is continued for 10 days, and on the 11th day the first nasopharyngeal swab is performed followed by a second one on the 13th day. If both tests turn out to be negative, the patient is considered healed and sent home with a certificate of healing.

The difficulty of screening lies in the fact that there are no symptoms directly attributable to COVID-19, apart from anosmia and ageusia, since fever or other flu symptoms may be attributable to other endemic diseases in Benin, such as malaria. Therefore, it is important to carry out specific tests, even though it is not always easy to reach the testing centres, wait for the slow turnaround of a non-computerized system and deal with the scarcity of tests, drugs and personnel in charge of the service.

‘Given the high rate of cases, the materials are not sufficient. In the specialised centres, there is an influx. The materials and beds are not enough.’—Hygienist, Male, 36 years old.

As for drugs, moreover, an interviewee points out how initially Chinese-imported chloroquine at 500 mg was administered in hospitals. However, once these stocks were finished, chloroquine was divided into 250-mg doses.

In fact, with the onset of COVID-19, Benin not only had the merit of tackling the scarcity of drugs and starting a local production of personal protective equipment, but also established low fixed prices to allow everyone to be able to purchase them without having to resort to the sector of the non-legal drug market, which is quite developed in Benin (Ouendo *et al.*, 2005; Dresse and De Baeremaeker, 2013).

‘[…] Still, the state has ensured that prices are kept at the same level in all pharmacies; the mask now I know it’s at 100 CFA (1 West African CFA franc = 0.0015 Euro), the chloroquine 500 mg was at 1000 CFA per pack’—Doctor, Male, 54 years old.

The scarcity of resources, as mentioned above, led to the occurrence of a situation universally shared by all countries which, in Benin as elsewhere, raised the bioethical question of the allocation of resources, locally dealt with prioritizing positive patients with comorbidities.

Certainly, the economic, technological, instrumental and organizational limits of the African countries, including Benin, represented an important bias that hindered and slowed down the response to the pandemic catastrophe, when compared with that of higher resource settings.

Regarding this, some interviewees pointed out that the application of the WHO directives is not uniform in all countries. This recalls a very significant bioethical question, namely that of the generalist normative universalism, which claims to apply norms and precepts in all the contexts uniformly, without considering the specific situations in which they must be applied (Pecchia *et al.*, 2020).

#### Traditional medicine/endogenous medicine

Benin is home to a traditional phytotherapeutic and herbal medicine [Although in this document the term ‘traditional medicine’ will be used as it is the official name used by the government, ‘popular’ or ‘endogenous’ medicine, in the sense of ‘products drawn from its own cultural fund, experienced by society as an integral part of its heritage, as opposed to exogenous knowledge perceived as elements of another system of values’ (Hountondji, 1994), is a more suitable name.] with such an ancient history and such a decisive value (Kpatchavi, 2011; Dresse and De Baeremaeker, 2013) that it is also recognized by the Beninese Ministry of Health, which has established a National Program of Pharmacopeia and Traditional Medicine, which it relies on for the treatment of various diseases through preparations that are more readily available and affordable (Benoist, 1989; WHO, 2019).

Also for the treatment and prevention of COVID-19, the remedies of traditional medicine were soon resorted to (i.e. API COVID-19 and EB+ COVID-19). However, this alternative treatment did not gain much interest from the healthcare authorities (Hountondji, 1994).

These remedies were different from the traditional rituals that are linked to local animism [such as the consultation of the Fâ (Fâ is a divinatory art traditionally practiced by the populations of the Gulf of Benin) and the consequent expiatory remedies] and were also used in some cases. These remedies were rather based on the use of the same natural principles on which modern medicine partially relies on, and could be a potential alternative in a context of scarce resources.

Certainly, the inherent risk in the use of traditional preparations, is that of the difficulty in controlling dosages, which could lead to dramatic consequences such as intoxication or death. Public health institutions would, therefore, benefit from scientific studies investigating the efficacy and safety of such traditional treatments. In fact, this use of local medicine could be an example of that necessary negotiation between normative universalism and situational contextualism, to be pursued with greater incisiveness.

‘Regarding this, there are a lot of herbal recipes that have a certain content of chloroquine within them. Traditional medicine should not be stigmatized, rather the active ingredients, their model of action on the body should be scientifically explored’—Doctor, Male, 62 years old.

‘Many use herbal medicines especially anything that is bitter; […] artemisia prevents coronavirus […]; others have used ginger, honey, lemon and turmeric, there is what they also call Kodo, they prepare it and drink it. […] [others] believe that hot teas also prevent the coronavirus.’

‘Consulting the Fa and expiatory rites to ward off bad luck. In the middle of the Abomey plateau in southern Benin, the epidemic news with skin rashes and chickenpox are giving rise to expiatory practices’.—Doctor, Male, 54 years old.

#### Perception of the disease

As regards the responsibilities of the government, the interviews conducted for this research reveal an excessive slowness in the communication of the test results, which evidently had consequences that cannot be underestimated, especially for COVID-19 positive patients and those who entered in contact with them, as well as a non-ideal management of people in quarantine.

With respect to the acceptance of measures related to the containment of the virus, many interviewees pointed out that travellers, who have been quarantined in hotels designated for this purpose, found themselves experiencing traumatic days, due not only to the uncertainty about their health, but also to the neglect of the health service that was reserved for them. In fact, the results of the tests were delayed and the quarantine for many people was extended from 14 to 21 days. Moreover, many patients (e.g. the elderly, pregnant women, etc.) needs were not taken into consideration.

In addition, some interviewees highlighted financial perplexities as regards the destination of public funds that could have been used for health emergencies and not for the hotels to quarantine travellers. According to some, in fact, the decision to close the airports would have avoided these long-standing difficulties.

Many interviews revealed the malaise experienced by these travellers confined to hotel sites ranging from feelings of frustration and discomfort to episodes of rebellion, sedated by the intervention of the police.

Furthermore, it should be said, in general, that the interviews revealed a significant difficulty of complying with containment measures, of the local population, particularly accustomed to shared sociability and sharing of daily rituals with the collectivity of the group (Lévy-Bruhl and Clare, 1966).

The difficulties of the imposed isolation and the sanitary cordons that reduced the supply of raw materials in the country and the consequent increase in prices caused a surgeon stress, which led to episodes of revolt and to the increase in domestic violence (Katana *et al.*, 2021). Furthermore, the forced abstention from work had the effect of increasing boredom and the significant increase in alcohol consumption.

What is surprising is that, although Benin has put in place a remarkable preventive strategy to increase its preparedness to manage the pandemic, it has never accompanied its security measures with relative legislative measures that guaranteed against any evasion, by punishing offenders.

In addition, Benin has not provided substantial support for workers who have been drastically affected by the isolation measures imposed by the pandemic. In particular, it is worth taking into consideration the informal economy sector which, in Benin, represents 97% of the gross domestic product and which was certainly among the most affected one by the economic and social consequences of the pandemic. This was underlined with insistence in the interviews along with proposals for direct support measures (through the distribution of food to the poor) and the reduction of taxes. The government has intervened, up to now, by offering economic support, in a single payment, to specific categories of workers considered most affected by the restrictive measures, which did not include workers in the informal sector (Stiftung, 2020).

Also particularly noteworthy is the sudden withdrawal by the state of all security measures, which gave way to a reliance on individual responsibility. This sudden step backwards, due to the balance between health imperatives and economic logic, received much criticism from the interviewees who are particularly confused by this behaviour. In fact, at the very peak of the pandemic, the state authorized local elections, allowed children to return to school and the faithful to access places of worship, and removed the sanitary cordon, keeping in place only the measures of hand washing and use of the mask.

The weakening of the restrictions by the government had an impact on individual attitudes to the extent that individuals were led to believe that the danger announced by the WHO had been overcome.

This sudden change in the management strategy of the pandemic, united to the scientific uncertainties about the virus itself and to the different interpretations of experts and scientists was included in the categories of ‘infodemics’ and ‘disinfodemics’ by the WHO and UNESCO (Hu *et al.*, 2020; Posetti and Bontcheva, 2020; Tangcharoensathien *et al.*, 2020; Younis, 2020), which, with this newly coined lemma, also defined the consequent psychological condition of confusion and uncertainty generated on the population, as confirmed by most of the interviewees.

The initial fear of the population resulted in spontaneous comparisons with other communicable diseases spread locally, such as Ebola or Lassa fever, and the finding of a lower mortality and symptomatology from COVID-19 helped corroborate the feeling of underestimation of the pandemic event.

In this regard, from the interviews it was possible to detect a particular caution shown by the Beninese authorities in the transmission of information, in particular in the dissemination of dramatic images that could have greatly affected people’s sensitivity. This opens up some interesting hermeneutical questions: the reasons behind this choice, whether it was for protection or to avoid unpredictable reactions by the frightened population, both of which would be related to a questionable state paternalism, are unknown.

‘There are ways to communicate and impact. There are possible steps to follow in communication, this being a very important element in the management of the pandemic crisis. When Westerners showed pictures of infected people undergoing treatment, there was general panic. But here, we don’t show any of that. There is nothing sensitive that the people are not afraid of. It is in communication that we must awaken people’s awareness of respecting barrier gestures. And that’s what I’m suggesting’. Anthropologist, Male, 39 years old

As Abel and McQueen (Abel and McQueen, 2021) argued, (dis)information overflow and political propaganda can make the situation more uncertain in respect to what public health measures should be accepted and followed. For future pandemics, there should be a more tailored approach according to which individuals should not be treated as objects. The compliance of individuals with public health measures depends on their proper communication and on the trust placed on the messenger. It is, then, clear how critical health literacy, by giving individual the ability to discriminate information, can play a crucial role in future pandemics and crises.

#### Sociocultural representations

Epidemic outbreaks reveal logics that mobilize values linked to sociocultural representations (Benoist, 2007; Massé *et al.*, 2011). The latter are likely to tip the field of social interpretations of epidemic risk into a regime of doubt but also of vulnerability in connection with biomedical discourse (Benoist, 2007; Fribault, 2015). These views are characteristic of the epistemological alignment of medical anthropology and refer to a dimension that goes beyond the limits of biomedical manifestations *stricto sensu* of epidemic outbreaks (Desclaux and Sow, 2015).

For this reason, as for the perception of the current pandemic, different orientations can be schematically identified, which have certainly been corroborated by the uncertainty of the state attitude:

(1) Adherence to the denier thesis
(2) Adherence to interpretations related to traditional culture

Each of the two orientations has specific variants.

The denialist orientation is based on the scarcity of epidemiological data relating to the contagion in the country and reports the aforementioned hypotheses of the alleged African immunity (genetic, personal, environmental) with respect to a disease that is attributed only to some populations, in particular to the Caucasian population. Opinions that the diagnosis or death from Coronavirus is unreliable and to be attributed to other diseases also contributes to denialism. This attitude of disbelief converges in the trivialization or underestimation of the pandemic that reaches its extreme with the adherence to full denial that, as it emerges from the interviews, seems to be very widespread in Benin.

‘However, when you watch a white man approach the natives in the market, you find that they automatically pull themselves together. This perception, linked to the fact that the coronavirus is a white man’s disease, causes users of the Dantokpa market to neglect each other […]’ Anthropologist, Male, 39 years old

This denialist interpretative vein, together with the still very intense pain of the colonial domination, leads to the consolidation of some theses, already present with the advent of HIV, including that of the anti-birth conspiracy, according to which, COVID-19 is a lie used as a demographic weapon to control the numerosity of the population (Alper *et al.*, 2021). This perspective matures in the background of the ‘clash of civilizations’ which, as literature and history confirm, has fuelled theoretical perspectives due to the ‘power of the imagination’ and the ‘power of persuasion’, typical of peoples who have suffered domination (Schultz and Lavenda, 2005).

A more moderate version of this orientation is the one that sees in the current situation a form of neo-colonialism for which the West is planning a plot with economic ends (to increase earnings by selling drugs and masks) or an ideological ‘scam’: fearing dramatic scenarios of the future spread of COVID-19 in Africa, the West wishes to show its ready aid to countries in difficulty, thus perpetuating its dominion.

‘The very behaviour of white people: we create things and then we come as a firefighter always for their own interests to the point that when there are serious problems, people trivialize: they say no, it’s an invention. The government and their sides. It’s not a real disease’. Epidemiologist, Male, 58 years old

‘Which support the hypothesis of a “conspiracy” around the imported masks’. Expert of Public health, Male, 60 years old

‘We also note the denial: For many, the disease does not exist; it is a worldwide scam’. Policeman, Male, 33 years old

The second line of interpretation stems from the traditional cultural background and does not only rely on the aforementioned therapeutic practices of traditional medicine, considering them more effective for African men than modern medicine, but also refers to animist spirituality. According to some, it would be possible to ascribe the aetiology of COVID-19 to the contemporary dissolution of morality that threatens an apocalyptic outcome if there is no change in human behaviour. Thus, consultations of the oracles were put in place, with the aim of researching the etiopathogenesis to calibrate the therapeutic-spiritual intervention based on it: alongside the herbal remedies, expiatory rites were celebrated, based on superimposition, typical of animist culture, between diseases and spells. In fact, according to this tradition, prevalent in the Fon cultural areas, spread throughout the country, the disease is typically considered the empirical manifestation of the breakdown of the physical–metaphysical order due to moral reasons or ‘envôutement’, i.e. jinxing (Dossi, 2015). These beliefs lead us to consider the sick person as the bearer of the evil that is incarnated in them and that intends to strike the physical world through them. This implies that the patient is in most cases looked at with suspicion and considered susceptible to contagion, even if the pathology from which they suffer does not suggest any type of biological transmissibility.

Compared with today’s COVID-19 situation, this stigmatization of the sick is clearly even more marked, since it is a communicable disease also from a biological point of view (Egrot, 2007). The stigma therefore leads to keeping positive people at a distance, for the fear of a contagion not only physical, but also metaphysical. The interviews report a total marginalization of the sick, in particular the elderly and the most vulnerable (for example those who have other comorbidities) to whom the law has imposed isolation, because they are considered particularly at risk, but also of healthcare workers and of Westerners who are considered to be more exposed to contagion.

Desocialization is also applied to positive suspects or those who have recovered from the virus to the point that the government has provided for the issuance of ‘recovery certificates’ for negativized patients who struggled to be readmitted into the social and work context. Stigmatization is one of the most dramatic social consequences of the pandemic, which brings with it the risk of avoiding being tested or self-reported for fear of social exclusion, making the commitment of individuals and the government to contain the contagion useless.

On closer inspection in a context such as Benin, the economic and social consequences of the pandemic far outweigh the health consequences (consider the increase in illiteracy due to the closure of schools, the difficulty of accessing essential health services due to the overcrowding of hospitals).

For these reasons, it seems worthy to investigate the singular circumstances for which the dramatic extent of the consequences exceeds that of the spread of the virus. It seems that this is an ideological problem: it is as if the fear of the virus had caused more serious consequences than the virus itself.

#### The emotion of fear

Fear has always been a philosophical, as well as a psychological word, on which many contemporary theorists have developed important reflections: from heuristics, theorized by Hans Jonas, according to whom profiling a terrifying picture could activate in individuals the principle of responsibility (Jonas, 1984) capable of promoting responsible actions to protect future generations, up to the reflections of the American philosopher Martha Nussbaum, a theorist of a social democracy based on skills, who modernly argues of the monarchy of fear (Nussbaum, 2019), of escaping fear from the emotional dimension of the private sphere, to enter the public scene, which also seems to occur during the so-called ‘COVID-19 era’.

In fact, it seems that fear has led governments, such as the Beninese one, to spread an initial alarmism and to take drastic measures, consistent with those of the ‘first world’ which, however, was experiencing a very different epidemiological situation. As a consequence, when the situation was compared with local data, it soon led to trivialization and denial with all the load of economic and social consequences mentioned above. Nussbaum, in this regard, observes that fear acts to cause alarmism and weakens capacity for judgement. Furthermore, as seen in the case of Benin, the fear of oppression, linked to historical resentment towards the West, has led to the identification of culprits, fuelling undemocratic attitudes. Even in this respect, Nussbaum argues that the wrath-blame mechanism is typical of fear. Nonetheless, as seen in the reported case study, the fear of contagion has led to the exclusion and stigmatization of individuals and groups, setting up what Nussbaum calls policies of disgust. On closer inspection, however, fear is a primitive, narcissistic and asocial emotion that is still causing more serious consequences in some countries than the spread of the virus itself. This arises when there is no adequate risk estimation.

Therefore, although it is extremely evident that science and its results must be universally usable, like health, which is a fundamental human right, it would seem appropriate to return to an effective risk assessment to better manage the consequences that derive from fear. This does not mean—as the Beninese government seems to have done—minimizing the current situation, particularly for already particularly vulnerable countries, but rather trying to close the knowledge gap through a greater diffusion of culture and scientific information that can allow a lucid and democratic analysis that activates responsible behaviours commensurate with specific situations, in compliance with that universality that is mindful of the particularistic and contextual differences that flow into the ethics of the situation. This means replacing fear with other emotions such as hope which, in Kantian terms, must be understood as a practical postulate and motor of action.

## CONCLUSIONS

This study reveals technical, political, ethical and sociological aspects of the preparedness to COVID-19 in a LRS, which, in their interconnection, show the importance of framing the issue with an interdisciplinary framework to offer a point of view that is complete and free from preconceptions. Furthermore, the study, although starting from the observation that the pandemic has put all countries on a universal level, namely that of the LRSs, leads to the conclusion that the hiatus among people and countries unfortunately still remains very strong: high-resource settings are struggling to find strategies for the sanitary containment of the virus, the rest of the world is suffering the consequences of a virus that has barely appeared on its side. Therefore, it seemed appropriate to re-investigate the reason for the spread of such dramatic economic and social consequences, in the face of a contained spread of the virus. It resulted that the ideology of individual and governmental fear has led to incorrect estimates of the risk that have been a harbinger of the aforementioned consequences. Ultimately, what emerges is that a generalized approach, even in the management of the COVID-19 pandemic, is always to be avoided because it leads to very serious consequences, such as those experienced in Benin. Rather, a management that is contextualized, situational and commensurate with the circumstance, in the enhancement of the diversities and differences allows an appropriate approach and avoids the universalism that is so general to result generic.

## Supplementary Material

daac105_suppl_Supplementary_Material

## Data Availability

The original text of the interviews is available by contacting the authors upon reasonable request.
