# Supplementary material for: COVID-19 preparedness and social dynamics in a Sub-Saharan Africa country, Benin
Source: Health Promot Int. 2022 Aug 24;37(4):daac105. doi: 10.1093/heapro/daac105 (PMC9400093; doi:10.1093/heapro/daac105)
Supplement: daac105_suppl_Supplementary_Material [file daac105_suppl_supplementary_material.docx]

Online Resource 1. A summary of all the COVID-19 related government provisions in Benin

| **Date** | **Public health measure implemented** | **Authority** |
| --- | --- | --- |
| 11 February | Health checkpoint for passengers at airport and land borders  Self-isolation of passengers coming from affected countries | Ministry of Health |
| 17 February | Entry ban on passengers coming from China | Government |
| 3 March | Toll free phone numbers to alert on suspicious cases or people coming from China bypassing border controls | Ministry of Health |
| 18 March | Limit entry and exit at land borders to goods only  Restrict entry visa to the country  Mandatory supervised 14-day quarantine for incoming people  Suspension of public events and unnecessary shops  Stocks of masks made available | Government |
| 23 March | Early Easter holidays  Sanitary cordon starting from 30^th^ March surrounding 12 cities  No movement in and out of the cordon is allowed, except for goods | Government |
| 7 April | Mandatory wear of mask inside the sanitary cordon | Government |
| 10 April | Schools, universities, nurseries, places of worship close for 1 month | Government |
| 14 April | Three cities are added to the sanitary cordon | Government |
| 27 April | Mandatory wear of mask nationwide | Government |
| 27 May | Reopening of bars, places of worship and resumption of circulation of public transport vehicles as of Tuesday, June 2, 2020 while respecting the barriers and official measures  Bars promoters and managers are required to ensure that the preventive measures enacted within them are observed, namely:  - the installation of a device for washing or sanitizing the hands  - compliance with the health safety distance of at least one meter between users  Religious leaders must, however, ensure that the wearing of masks, hand washing and the respect of the health safety distance of at least one meter between people are observed. They must also, for those who practice it, prescribe the suspension of the liturgical gesture of peace or of the hugs during the celebrations. | Government |

Online Resource 2. A summary of the hierarchy of the nodes (themes) along with their description.

| Node | Subnode | Subsubnode | Subsubsubnode | Description |
| --- | --- | --- | --- | --- |
| Disease management | Clinic | Limits of the public health system | Disinformation | Management of the Covid-19 pandemic by the health system, from a clinical, but also ethical and psychological point of view, with its limitations, many of which related to poor communication, and its strengths. |
|  |  | Prevention |  |  |
|  | Ethical |  |  |  |
|  | Psychological |  |  |  |
| Governmental measures | Easing of measures |  |  | Government management of the pandemic from a health (biomedical equipment, hygiene), economic (sanitary cordons), political point of view |
|  | Economic issues |  |  |  |
|  | Increased hygiene |  |  |  |
|  | Increasing of MD |  |  |  |
|  | Increasing of Police |  |  |  |
|  | Regulatory gap |  |  |  |
|  | Sanitary cordon |  |  |  |
| Isolation | In communities |  |  | Issues emerged from the extent of the isolation of both travellers entering Benin and within the communities. |
|  | In hotel (travellers) | Financial problems |  |  |
|  |  | Organizational problems |  |  |
|  |  | Rebellions |  |  |
|  |  | Uneasiness |  |  |
| Perception | African biological immunity |  |  | Theories formulated by the local population to interpret the virus, its causes (cultural and historical), the sensations experienced in respect to the pandemic and the measures requested by national and international authorities. Consequences and effects of the interpretations and emotional states reported. |
|  | Anger |  |  |  |
|  | Comparison with other diseases |  |  |  |
|  | Denial |  |  |  |
|  | Difficulty in using masks |  |  |  |
|  | Difficulty of isolation in communities |  |  |  |
|  | Difficulty of physical distancing in public places |  |  |  |
|  | Distress | Consequences |  |  |
|  | Distrust in pharmacology |  |  |  |
|  | Distrust of WHO clinical trials |  |  |  |
|  | Environmental condition |  |  |  |
|  | Fear | Stigmatization |  |  |
|  | Uncertainty |  |  |  |
|  | Underestimation |  |  |  |
| Pharmacological treatment |  |  |  | Therapeutic strategy used in Beninese health facilities |
| Screening | Call centre |  |  | Local strategies for the screening of the virus |
|  | Laboratory analysis |  |  |  |
| Traditional medicine | Limits of traditional medicine |  |  | Risks and benefits of therapeutic practices (preventive and curative) related to local traditional medicine. |
